# Impact Assessment of Urethral Meatus Morphology and Penile Biometry in Transurethral Prostate and Bladder Surgery

**DOI:** 10.1155/2017/6321702

**Published:** 2017-02-19

**Authors:** Rodrigo Ribeiro Vieiralves, Paulo Henrique Pereira Conte, Eduardo Medina Felici, Nádia Cristina Pinheiro Rodrigues, Tomás Accioly de souza, Francisco J. B. Sampaio, Luciano Alves Favorito

**Affiliations:** ^1^Department of Urology, Lagoa Federal Hospital, Rio de Janeiro, RJ, Brazil; ^2^Department of Technology Information, Medical Sciences School, Rio de Janeiro State University, Rio de Janeiro, RJ, Brazil; ^3^Urogenital Research Unit, Rio de Janeiro State University, Av. 28 de Setembro, 77 Fundos Vila Izabel, 20250-050 Rio de Janeiro, RJ, Brazil

## Abstract

*Objective*. To analyze the penile and urethral meatus biometry and its correlation with meatoplasty during endoscopic resections. We also propose a new classification for urethral meatus morphology.* Materials and Methods*. We prospectively studied 105 patients who underwent prostate and bladder transurethral resections. We performed standardized measurement of penile and urethral meatus biometry followed by penile photo in the front position. The need to perform meatoplasty or dilatation during resectoscope introduction was registered. Data were analyzed comparing the correlation between two groups: without intervention (Group A) and with intervention (Group B).* Results*. We observed in Group A and Group B, respectively, the average length of urethral meatus of 1.07 cm versus 0.75 cm (*p* < 0.001) and average width of urethral meatus of 0.59 cm versus 0.38 cm (*p* < 0.001). Considering the morphology of the urethral meatus, we propose a new classification, in the following groups: (a) typical; (b) slit; (c) point-like; (d) horseshoe; and (e) megameatus. The point-like meatus was the one that most needed intervention, followed by the slit and the typical meatus (*p* < 0.001).* Conclusions*. Point-like and slit-shaped urethral meatus, as well as reduced length and width of the urethral meatus, are the determining factors.

## 1. Introduction

Benign prostatic hyperplasia (BPH) is one of the most common diseases in men, with progressive incidence according to age [1]. BPH leads to lower urinary tract symptoms (LUTS) due to bladder outlet obstruction [1]. LUTS have an important impact on quality of life by interfering directly in daily activities and sleep patterns. According to the clinical presentation, there are several treatment options for BPH such as watchful waiting, pharmacological management, and surgical treatment. Minimally invasive treatment through transurethral resection of the prostate (TURP) is considered the gold standard for surgical therapies for prostates up to 80 g. Other treatments such as open prostatectomy are reserved for larger prostates [2]. Malignant neoplasm of the bladder occupies the seventh place in the ranking among the most common cancers in men [3]. Invariably, the initial approach requires transurethral resection of the bladder (TURB), which is the key to diagnosis and initial treatment.

Prostate and bladder TUR have several possible complications, such as bleeding, prostatic capsule perforation, bladder perforation, post-TUR irrigating fluid syndrome complications (for monopolar resection only), and urethral stricture [4, 5]. One of the factors involved in urethral stricture after TUR is the narrow diameter of the urethra [6]. One of the critical factors when introducing the resectoscope is the size of the urethral meatus (UM) [7]. When the UM is too narrow, not allowing the passage of the device, the surgeon can first try introduction guided by a urethral shutter. When this is not possible, meatoplasty (surgical opening of the urethral meatus through simple incision) will be required.

Previous studies analyzing the anatomy of the UM are scarce in the literature. Walton examined the UM in 59 patients but did not correlate it with other measures of the penis [7]. The UM size in boys and its correlation with growth are described in the literature, as well as the UM biometry applied to hypospadias surgery [8–12]. However, the analysis of the structure of the UM and its correlation with penile biometry during TUR is unprecedented in the literature.

We hypothesize that the UM anatomy and penile biometry could be involved in difficult passage of the resectoscope during transurethral surgery. We tested this hypothesis by evaluating the structure of the UM and making measurements of the penile shaft and urethral meatus. The aim of this study was to analyze the penile and the urethral meatus biometry and its correlation with the need for meatoplasty during prostate and bladder endoscopic resections. We also propose a new classification for urethral meatus morphology.

## 2. Materials and Methods

The experimental protocol described here was approved by the ethical committee for human experimentation of our university. This study was carried out in accordance with the ethical standards of the hospital's institutional committee on human experimentation.

From October 2014 to April 2016 we studied 105 patients who underwent TUR of the prostate or bladder. Patients with urethral stricture, previous urethral surgery, or use of Foley catheters were excluded from the study. Biometry evaluation of 105 patients was performed by a single examiner in a standardized manner.

We measured penile shaft and the urethral meatus (width, circumference, and length of the penis; width and length of the urethral meatus) with the aid of an anthropometric ruler, supported in the dorsal region of the flaccid penis, at maximum traction, depressing the pubic fat against the pubic bone. We therefore measured the penis length in maximum traction, equivalent to penis length during erection. The penile width and circumference were also measured with the penis held in maximum traction. For urethral meatus measurements, compression of the lateral axis of the glans was performed at the level of the glans corona. This compression was the mildest possible to ensure minimal opening of the UM, allowing measurement of its length and width and classification of meatus types.

**Figure 1:**
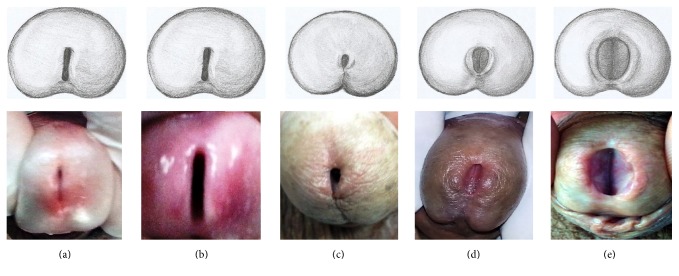
Urethral meatus morphology: the figure shows a schematic drawing (top) and examples of pictures of patients in our study (lower) with the types of urethral meatus found: (a) typical meatus; (b) slit meatus; (c) point-like meatus; (d) horseshoe meatus, and (e) megameatus.

For the evaluation of the UM morphology, immediately after the evaluation of UM biometric parameters, still using the standard technique reported, a penile picture was taken in front position. The photos were filed in the record of each patient in digital format. At the end of the study, the photos of the 105 patients were analyzed by three different investigators to formulate a classification in five different types of urethral meatus (Figure 1). All 105 patients fit into one of these categories.

In order to standardize the TUR so that the technique while introducing the resectoscope was always the same, all patients were operated on by the same surgeon. We used an Olympus® 26Fr resectoscope operating in continuous flow. The electrodes used were of the handle type. No buttons were used in our study. An Olympus bipolar plasma generator was employed, which utilizes saline for irrigation. After the procedure, all patients remained with a 22 FR Foley catheter and continuous bladder irrigation with saline solution for at least 24 hours. All data collected were organized through a standardized form, containing patient identification, relevant aspects of the procedure, and medical history. The withdrawal photos were stored in digital format and saved in files individually identified by the initials of the patient's name, as well as by the number of the form.

### 2.1. Statistical Analysis

Data from 105 patients were compared by evaluating the correlation of two distinct groups: without intervention (Group A) and with intervention (with urethral dilator use or submitted to meatoplasty, Group B). We also compared the mean measures found according to five different groups according to morphological findings. The hypothesis that the studied variables (penile length, width and circumference as well as the width, length, and morphology of the urethral meatus) would have an association with greater chance to use urethral dilator or perform meatoplasty during transurethral resection was tested on the basis of the obtained values. The measurement data were analyzed by the *t*-test (stratified analysis between two groups) and ANOVA (when comparing the means of all five morphological groups). The urethral meatus morphology categorical data versus intervention need were analyzed with Fisher's exact test (*p* < 0.05) [13].

## 3. Results

We performed 65 TURP, 37 TURB, and 3 resections of the prostate and bladder. The average length, circumference, and width of the penis and the average length and width of the UM can be seen in Table 1.

There was urethral 28Fr dilator use in 15 patients (14.2%) and need for meatoplasty in 5 patients (4.7%). Comparing the two groups, we identified a statistically significant difference for mean UM length, of 1.07 cm/SD = 0.31 (Group A) versus 0.75 cm/SD = 0.14 (Group B) and mean UM width of 0.59 cm/SD = 0.20 (Group A) versus 0.38 cm/SD = 0.07 (Group B) (*p* < 0.001). For the average penile length, penile width, and penile circumference, there were no differences between groups.

Regarding UM morphology, we propose a new classification into five groups according to the UM form. All patients fit into these new categories. The groups in order of frequency were (a) typical meatus; (b) slit meatus; (c) point-like meatus; (d) horseshoe meatus; and (e) megameatus. All types of UM found in our study can be seen in Figure 1.

The one-way analysis of variance and overall comparison of the morphology groups and different types of UM biometric findings indicated that the differences found in length and width of the UM correlate with the type of meatus; that is, different UM morphologies exhibit statistically significant differences in UM length and width measurements (*p* < 0.001). In contrast, the differences found in penile biometrics were not associated with the morphology of the UM: length (*p* = 0.243); width (*p* = 0.842); and circumference (*p* = 0.407).

Similarly, when comparing the different types of meatus according to the need for intervention, we observed that the behavior of the different UM morphologies required significantly different intervention rates (Table 1). The point-like meatus was the one that most needed intervention (12 cases: 57%), followed by the slit meatus (6 cases: 19%) and the typical meatus (2 cases: 5%), in all cases with statistical significance (*p* < 0.001).

Regarding UM morphology, we found significant differences in length and width of the UM correlated with the type of meatus. For the length, we found that the point-like meatuses were significantly smaller than the typical (*p* < 0.001), slit (*p* < 0.001), horseshoe (*p* = 0.001), and megameatus types (*p* = 0.021). The slit meatus was significantly smaller only than the megameatus (*p* = 0.041). For the UM width, we found that point-like meatus was significantly smaller than the horseshoe (*p* = 0.025) and megameatus (*p* = 0.013). The slit meatus was significantly smaller than the typical (*p* = 0.002), horseshoe (*p* = 0.003), and megameatus (*p* = 0.015).

## 4. Discussion

Knowledge of ethnic and individual variations in penis size is of great assistance in the diagnosis and treatment of various conditions in childhood and adulthood [14]. Several studies have analyzed penile biometrics in children and adults, allowing the development of nomograms that assist diagnosis and prevent misdiagnosis of micropenis, for example, [15–17]. However, there are no reports in the literature that relate the measures of the penis to the need for transurethral resection. We observed that the penile length, width, and circumference had no influence on performing meatoplasty or dilator use during the introduction of the resectoscope. We also observed there was no association between the length, width, and circumference of the penis and the urethral meatus structure. This fact shows the irrelevance of the penile biometric findings regarding endoscope manipulation in the retrograde urinary tract.

In contrast, the results found indicate that UM is the most important aspect when performing endoscopic retrograde manipulation of the urinary tract because its metrics can limit the introduction of endoscopic devices. Several studies have analyzed the position of the urethral meatus in children and adults without penile abnormalities in order to justify the need for hypospadias surgery, with advancement of the meatus in distal hypospadias [12, 18]. Walton examined the urethral meatus of 59 patients and found no correlation between measures of the penis and the UM shape [7].

We noted an absence of studies analyzing the measures of UM in patients undergoing transurethral surgery. We also observed that previous classification and analysis of the shape and type of UM with respect to transurethral surgery are absent in the literature. A fact of great interest during this study is that all patients could be grouped into one of the five UM categories of our morphological classification. We identified different behaviors during the handling of these five meatus types, revealing the clinical applicability of this classification system. From what has been shown, we believe that this classification represents an anatomical reality and will be useful in future studies involving UM.

In our statistical analysis, we observed that smaller UM widths and lengths are associated with the greatest chance for meatoplasty or dilator use. This information is intuitive, but without previous evidence. Regarding morphology, point-like meatus was the one with the smallest mean measures (length = 0.7 cm and width = 0.5 cm), while the horseshoe meatus and especially the megameatus had the largest mean measures (length = 1.1 cm and width = 0.63 cm/length = 1.86 cm and width = 1.03 cm, resp.).

The typical, slit, and point-like meatus required dilatation or meatoplasty during TUR. The typical meatus required intervention only in 5% of the cases, but the slit and point-like meatus need intervention more frequently. The 21 patients with point-like meatus required intervention in more than 50% of the cases and 60% of meatoplasty procedures were done in patients with point-like meatus. The point-like meatus had smaller length than the other types and smaller width than the horseshoe and megameatus. The slit meatus length was smaller only than megameatus, but the slit meatus widths were smaller than the others, except the point-like meatus. The horseshoe meatus and megameatus were the only types that required no intervention during TUR and had the largest lengths and widths. Although these two types of meatus showed low incidence, this information may be of clinical interest.

Therefore, a concept that arises from the analysis of these data is that during TUR, if the patient has a point-like meatus, the chance of the resectoscope passing without intervention will be smaller, so knowledge of the different types of UM may be of great aid to the urologist preoperatively, allowing informing the patient about possible meatoplasty, a procedure that is associated with meatus stenosis [6]. We found difficulty in the introduction of the resectoscope in only 19% of cases, but we do not have stenosis information about patients undergoing meatoplasty because of our short follow-up. An interesting study where patients underwent urethrocystography before and after urological instrumentation showed that 17% of patients had some degree of urethral stricture. However, that study does not describe any morphological parameters of the penis or UM [19].

The main limitations of our study are (1) the lack of a more accurate method for making measurements of the penis and UM, although the fact that the measures followed standards already established in the literature [20] and were performed by a single examiner decreases the chance of misinterpretation of the measurements and (2) the extended follow-up needed to evaluate the occurrence of UM stenosis.

## 5. Conclusions

When performing prostate and bladder TUR, there are factors associated with a higher chance of intervention (meatoplasty or dilatation). Point-like UM is a determining factor. The measures of the penis itself did not influence the need for intervention. Thus, through a prior assessment of the urethral meatus by physical examination, the urologist can predict the need for meatoplasty, providing more precise information to the patient.

## Figures and Tables

**Table 1:** The table shows the relationship between the type of urethral meatus and the measures of the meatus and penis in centimeters. We can also observe the relationship between the type of urethral meatus and the need of intervention. Last line *p* values statistically shows the biometric differences when comparing the different types of meatus. The intervention *p* value statistically demonstrates the meatus type intervention ratio differences significance. ML = length of the urethral meatus; MW = width of the urethral meatus; PL = penile length; PW = penile width; PC = circumference of the penis; MP = meatoplasty.

Meatus	ML (M/SD)	MW (M/SD)	PL (M/SD)	PW (M/SD)	PC (M/SD)	Intervention		
						Total	Dilator	MP
Typical 42 (40%)	0.5 to 2.0 (1.07/0.30)	0.3 to 1.5 (0.6/0.21)	8 to 15.5 (12.0/1.89)	2.5 to 4.6 (3.5/0.47)	9 to 14.4 (11.5/1.35)	2 (5%)	2 (100%)	0 (0%)
*Slit31 (29%)*	0.7 to 1.5 (1.0/0.21)	0.3 to 0.9 (0.4/0.15)	7 to 15.3 (11.1/2.16)	2.5 to 4 (3.4/0.49)	7.8 to 15 (10.9/1.70)	6 (19%)	4 (66%)	2 (33%)
*Point-like21 (20%)*	0.5 to 1.1 (0.7/0.16)	0.3 to 0.9 (0.5/0.17)	9 to 16 (11.9/1.73)	3 to 4.5 (3.5/0.39)	9 to 12.7 (10.8/1.0)	12 (57%)	9 (75%)	3 (25%)
*Horseshoe8 (7%)*	0.9 to 1.5 (1.1/0.22)	0.4 to 0.8 (0.6/0.11)	8.5 to 15.5 (11.3/2.37)	3 to 4.6 (3.6/0.58)	9.2 to 14.5 (11.2/1.93)	0 (0%)	0 (0%)	0 (0%)
*Megameatus3 (3%)*	1.5 to 2.1 (1.8/0.32)	0.9 to 1.2 (1.0/0.15)	9 to 12 (10.5/1.50)	3 to 4 (3.5/0.51)	9 to 12 (10.8/1.60)	0 (0%)	0 (0%)	0 (0%)
*Total105 (100%)*	0.5 to 2.1 (1.0/0.31)	0.3 to 1.5 (0.5/0.20)	7 to 16 (11.7/2.0)	2.5 to 4.6 (3.4/0.47)	7.8 to 15.0 (11.1/1.50)	20 (19%)	15 (75%)	5 (25%)

*pvalue*	*p* < 0.001	*p* < 0.001	*p* = 0.2433	*p* = 0.8423	*p* = 0.4067	*p* < 0.001		

## Abbreviations

BPH: Benign prostatic hyperplasia
LUTS: Lower urinary tract symptoms
TUR: Transurethral resection
TURP: Prostate transurethral resection
TURB: Bladder transurethral resection
UM: Urethral meatus
SD: Standard deviation
Cm: Centimeters
Fr: French.
